# Sustainable Development Strategies for RIS-Assisted Mobile Networks

**DOI:** 10.3390/s26103243

**Published:** 2026-05-20

**Authors:** Anwar Hassan Ibrahim

**Affiliations:** Department of Electrical Engineering, College of Engineering, Qassim University, Buraydah 51452, Saudi Arabia; an.yagoob@qu.edu.sa

**Keywords:** Internet of Things (IoT), mobile networks, network performance, Reconfigurable Intelligent Surface (RIS), simulation program, sustainability, sixth generation (6G)

## Abstract

The transition toward environmentally sustainable 6G networks requires mitigating the high-power consumption of traditional active base stations and relay nodes currently used to overcome signal path loss. This paper introduces Reconfigurable Intelligent Surfaces (RIS) as a paradigm-shifting, inherently passive alternative that alters the wireless propagation environment without requiring power-intensive radio frequency (RF) chains. Rather than focusing solely on spectral efficiency, this research aims to maximize Energy Efficiency (EE) to achieve a critical equilibrium between network performance and power consumption. MATLAB-based analytical models demonstrate that received signal power scales quadratically with the number of reflecting elements via constructive interference. Furthermore, systematic evaluations reveal that a 64-element RIS panel imposes a negligible hardware load consuming merely 0.005 Watts per element, offering a highly sustainable alternative to the massive transmit power (up to 40 dBm) frequently required by unassisted networks in noisy environments. By defining a mathematical “Green Operating Point,” this study demonstrates that integrating lightweight RIS panels significantly enhances Signal-to-Noise Ratio (SNR) and data rates, steering next-generation telecommunications toward highly sustainable, low-power operations.

## 1. Introduction

Cellular infrastructure power consumption must be fundamentally reevaluated in light of the paradigm change towards environmentally friendly 6G. The deployment of extra active base stations (BS) or power-intensive relay nodes is a major component of traditional approaches for overcoming severe path loss and obstructions. Reconfigurable Intelligent Surfaces (RIS), on the other hand, provide a transformative, passive-by-design substitute. Network operators can control the wireless propagation environment without using active radio frequency (RF) chains at the reflecting surface by carefully placing flat arrays of metamaterial elements.

Sustainable RIS Integration: Balancing Performance and Power in Next-Generation Mobile Systems is the main academic challenge. We must create an optimization problem focused on Energy Efficiency (EE), which is the ratio of the feasible sum-rate to the overall system power consumption, to thoroughly assess this balance [1,2].

In contrast to traditional Pareto-based multi-objective optimization techniques that offer a tradeoff surface between Energy Efficiency (EE) and Spectral Efficiency (SE), this study presents a useful operational framework focused on sustainability called the “Green Operating Point” (GOP). Under practical hardware power limits, the GOP finds a special operating threshold that maximizes bits-per-Joule. Additionally, by adding volumetric energy usage measures for fixed-payload IoT broadcasts, this work expands on traditional EE analysis.

RIS-assisted communication is seen to be a viable strategy for enhancing coverage and energy efficiency in upcoming wireless networks. However, channel conditions, deployment geometry, hardware limitations, control overhead, and phase configuration accuracy all affect its usefulness. Therefore, rather than portraying RIS as a universally better solution, this study assesses RIS-assisted transmission from a sustainability-oriented perspective.

The rest of this document is structured as follows: The history and relevant research on RIS-assisted sustainable communications are reviewed in Section 2. Strategies for allocating green resources are covered in Section 3. Integration with edge computing and Cell-Free Massive MIMO is presented in Section 4. Resilience and coverage improvement are highlighted in Section 5. The simulation model and mathematical framework are developed in Section 6. The outcomes and practical ramifications are covered in Section 7. Lastly, the contributions and conclusions are presented in Section 8 and Section 9, respectively.

## 2. The Research Gap

The use of Reconfigurable Intelligent Surfaces (RIS) to increase coverage and raw Spectral Efficiency (SE) in 5G-Advanced and 6G networks has been extensively studied; however, most of these studies rely on brute-forcing signal strength to overcome severe mmWave and sub-THz route loss. Determining dynamic, sustainable operating boundaries for these networks is still a crucial gap.

In particular, the literature currently in publication lacks a thorough mathematical framework that pinpoints the precise (Green Operating Point) the point at which attempting to achieve a slightly higher Signal-to-Noise Ratio (SNR) causes disastrous declines in total Energy Efficiency (EE). Additionally, the volumetric energy trade-offs (total battery drain over time) necessary for sustainable IoT operations in high-noise situations are often ignored in existing studies.

Few RIS studies offer an operationally deployable threshold for sustainable network control, nor do they measure volumetric battery-energy consumption for fixed IoT payload transmissions. Instead, existing RIS research primarily maximizes either spectral efficiency or instantaneous energy efficiency.

While RIS-assisted sustainable wireless communications has advanced significantly, the majority of recent research focuses on optimizing instantaneous Energy Efficiency (EE), Spectral Efficiency (SE), or throughput through joint beamforming, AI-driven optimization, or sophisticated architectures like STAR-RIS and Active RIS. For instance, Huang et al. established a fundamental EE framework by proposing joint transmit power and RIS phase-shift optimization for EE maximization in RIS-assisted systems. Using deep learning, reinforcement learning, and STAR-RIS-based resource management for 6G IoT sustainability and improved QoS in dynamic settings, more recent studies have expanded this approach. Nevertheless, despite these developments, the research now in publication hardly ever defines an operationally deployable sustainability threshold that specifically determines the highest bits-per-Joule operating point under practical hardware power restrictions. Additionally, little is known about the volumetric battery-energy consumption needed to send a fixed-size payload, which is a crucial statistic for sustainable IoT and battery-limited devices. To close the gap between theoretical EE optimization and real-world sustainable network operation, this work presents the Green Operating Point (GOP) paradigm.

## 3. Background

In the development of mobile networks beyond 5G (B5G) and 6G, Reconfigurable Intelligent Surfaces (RIS) have become a fundamental and disruptive technology. In order to produce a smart, adaptable radio environment, RIS is made up of a variety of passive, electronically controlled reflecting elements that can dynamically alter the propagation of electromagnetic waves [3,4]. RIS elements offer a very sustainable substitute for conventional relay systems since they do not require active power amplifiers to transfer signals. RIS significantly reduces the total energy consumption of wireless networks while smoothly increasing coverage by intelligently reflecting signals to get around obstacles or fortify links [5]. Comprehensive surveys such as Wu and Zhang (2020) [6] have highlighted RIS as a transformative enabler for beyond 5G and 6G networks, identifying energy efficiency and channel estimation as key challenges [7].

Reconfigurable intelligent surfaces, or RISs, have emerged as a promising technique to enhance wireless communication by deftly modifying the propagation environment. RISs, which are composed of several passive reflecting components, can be dynamically controlled to steer and focus radio waves under challenging propagation conditions, expanding coverage and capacity [8].

Severe coverage dead zones are produced in conventional dynamic rerouting and self-healing networks when a line-of-sight (LoS) link is lost because of a node failure or a new physical barrier. By serving as a programmed mirror, RIS lessens this. The network can quickly adjust the RIS phase shifts to create a robust, alternate non-line-of-sight (NLoS) path in the event that a major path is disrupted, guaranteeing continuous service without the need for redundant active base stations [9].

Using an infinite number of reflecting elements, Wu and Zhang (2020) [6] examined the asymptotic performance of IRS-based passive beamforming and compared it to conventional active beamforming and relaying. According to our simulations, an IRS-assisted MIMO system greatly reduces the number of active antennas and RF chains needed while maintaining the rate performance of a conventional massive MIMO system. Additionally, we gained useful knowledge for the best possible use of IRS technology in next-generation wireless systems [8,9].

Additionally, emphasizes the critical role that machine learning (ML) and artificial intelligence (AI) play in optimizing RIS-based beamforming to address difficult engineering problems while guaranteeing sustainability and energy efficiency in NTN operations. Their work highlights the importance of RISs in realizing the full potential of NTNs and developing next-generation communications by placing them as crucial enabler for the future of wireless systems.

The anti-jamming and physical layer security for RIS has become an essential anti-jamming tool as wireless networks confront more complex security threats. RIS panels maximize the Signal-to-Jamming-plus-Noise Ratio (SJNR) for authorized users while successfully nullifying hostile jamming signals by constantly adjusting their reflecting elements In order to ensure secure space-air-ground integrated networks (SAGIN), recent studies show that putting RIS aboard Unmanned Aerial Vehicles (UAVs) can effectively block powerful jamming attacks, including those coming from Geostationary Earth Orbit (GEO) satellites [10]. Additionally, RIS is being actively combined with reinforcement learning algorithms to continuously adapt to and silence smart jammers in real time [11].

Hardware Longevity and Sustainable Maintenance: An RIS network’s physical lifespan is part of its resilience. RIS panels produce less heat and undergo far less hardware degradation because they depend on passive reflecting elements instead of energy-intensive radio frequency (RF) chains and active power amplifiers. Long-term sustainability is aided by this passive nature, which significantly lowers maintenance needs and increases network infrastructure’s operating lifespan [12].

## 4. Sustainable Green Resource Allocation

The significant increase in energy efficiency (EE) that RIS integration provides is a major motivator for modern telecommunications. In comparison to systems that use on standard multi-antenna amplify-and-forward (AF) relays, foundational studies have shown that simultaneously optimizing transmit power allocation together with the phase shifts in RIS reflecting elements offers much greater EE [13]. While adaptive beamforming concentrates signal energy precisely where it is required, the passive nature of RIS elements reduces hardware energy losses. The raw transmission power needed at the base station or by the user equipment is decreased by this targeted wave alteration. Additionally, combining user-side power regulation with RIS reflective beamforming in uplink user-centric networks has proven to be very successful in maximizing spectral efficiency while preserving mobile users’ limited battery capacity [14].

In the context of Reconfigurable Intelligent Surfaces (RIS), Green Resource Allocation focuses on optimizing Energy Efficiency (EE) rather than just raw speed (Spectral Efficiency). We cannot just throw an infinite number of RIS elements at a network and transmit power; instead, the power model that adds additional RIS parts linearly increases power consumption. The goal of green resource allocation is to identify the mathematical “sweet spot” that minimizes the overall energy footprint (measured in bits per Joule) while delivering the necessary data rates.

**Figure 1:**
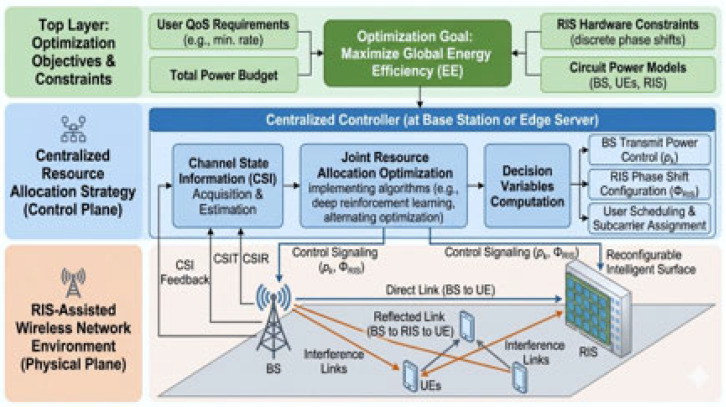
Complete system framework for RIS Green Resource Allocation.

The entire RIS Green Resource Allocation system framework is shown in Figure 1, which shows how many of the ideas we’ve covered work together to maximize energy efficiency. There are three logical stages in the diagram.

The technology minimizes the network’s overall energy footprint while maintaining a high quality of service by jointly optimizing these variables within an iterative algorithm stated with system scenario and channel model, joint resource allocation variables.

## 5. Integration with Cell-Free Massive MIMO and Edge Computing

**Figure 2:**
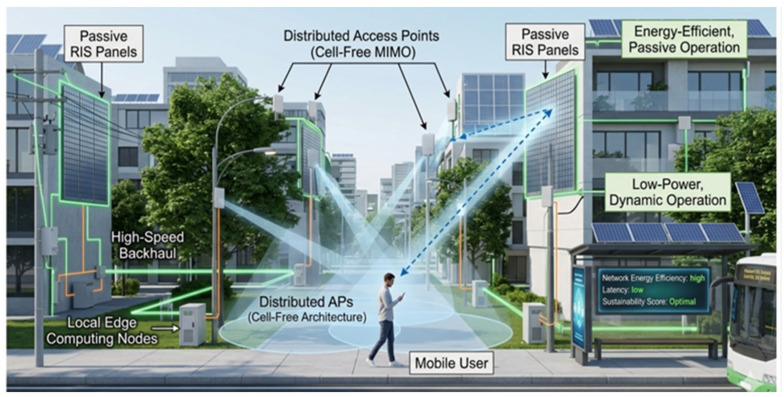
Sustainable RIS Assisted Cell Network.

Researchers are actively investigating RIS-aided cell-free massive MIMO systems to sustainably satisfy the enormous capacity requirements of 6G. The Sustainable RIS-Assisted Cell Network is depicted in Figure 2. Although massive MIMO significantly increases network capacity, a significant disadvantage is that active access points (APs) have considerable power consumption, especially during times of low load. By combining RIS activities with AP hibernation or sleep modes, recent green networking techniques reduce this. Networks can achieve significant EE gains by dynamically shutting off redundant APs and depending on RIS to maintain network coverage and quality of service [15]. In order to combat the multiplicative fading effects of long-distance links and maintain a highly energy-efficient footprint over dispersed cell-free deployments, some state-of-the-art systems even employ “active RIS” with little power amplification [16].

Active RIS may outperform passive RIS under extreme double-fading situations at the expense of additional energy costs, whereas passive RIS offers greater EE in moderate-noise environments due to less hardware power usage.

Additionally, combining RIS with Mobile Edge Computing (MEC) is advancing sustainable network computing. RIS-enabled MEC networks automatically divert signals to reduce the total energy consumption of users delegating computationally demanding tasks to edge servers by using Non-Orthogonal Multiple Access (NOMA) protocols [17].

## 6. RIS Network Resilience and Extended Coverage

Accessibility, hardware longevity, and network resilience are all included in sustainable development. Hardware wear and tear is reduced by the passive design of RIS components. By reducing the network’s life-cycle material and manufacturing footprint, the absence of active moving parts or high thermal output increases operational life and lowers maintenance requirements, hence indirectly promoting sustainability [8]. Additionally, within the framework of 6G “resilience-by-design,” RIS provides essential dynamic reconfiguration capabilities. RIS can adjust to maintain acceptable service levels in the event of malicious jamming assaults or localized infrastructure failures without the need for quick, resource-intensive physical operations [18]. Finally, by incorporating RIS into High-Altitude Platforms (HAPs) and Non-Terrestrial Networks (NTNs), reliable connection can be extended to underserved or distant locations without the exponential power costs usually associated with large-scale aerial deployments [8]. Networks face significant propagation issues as wireless standards move toward millimeter-wave (mmWave) and Terahertz (THz) bands to reach huge data speeds. High-frequency signals have severe route loss and are difficult to pass through physical barriers.

**Figure 3:**
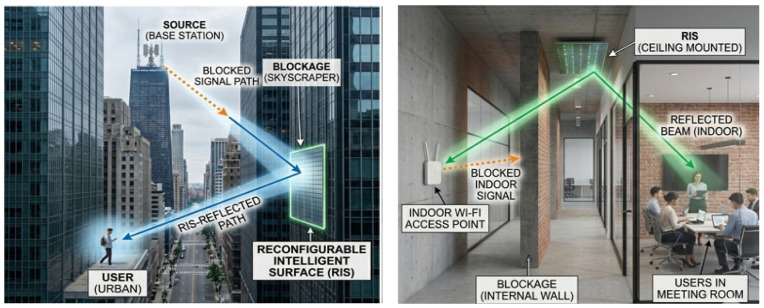
Urban and Indoor Blockage overcomes.

The RIS resolves the crucial mmWave obstruction issue to get around interior and urban barriers. As illustrated in Figure 3, network operators can strategically place passive RIS panels on building facades or interior walls to fill urban blind spots rather than using a large number of power-hungry active small cells. These surfaces pick up signals and reroute them around obstructions and corners. According to research, strategically positioning RIS in urban canyons and dense indoor spaces can ensure high-speed data rates and significantly increase mmWave coverage while maintaining remarkably low deployment costs and energy consumption [9].

This is the point at which Reconfigurable Intelligent Surfaces (RIS) become a truly disruptive technology for 5G-Advanced and 6G networks, rather than just a clever mathematical trick. We must examine the radio frequencies being used to comprehend why RIS is so important in this situation. In order to achieve enormous bandwidth, modern networks are shifting toward millimeter-wave (mmWave) and terahertz (THz) frequencies. Similar to visible light, these high-frequency signals go in straight lines and are readily obstructed by walls, trees, buildings, and even human beings.

Researchers are merging RIS with High-Altitude Platforms (HAPs) and UAVs to provide sustainable connectivity to remote, rural, or disaster-affected areas through Non-Terrestrial Networks (NTNs) and Aerial Expansion. The flight time of traditional drone-based active relays is significantly limited by their enormous battery consumption. However, UAVs can remain in the air for a lot longer when they are outfitted with lightweight, passive RIS panels. With a minimum carbon and energy footprint, these Aerial RIS (ARIS) nodes may dynamically reflect signals from far-off ground or satellite stations directly to underserved consumers, greatly expanding the network’s geographic reach [19,20].

## 7. Mathematical Core of Sustainable Network Development

The Green Curve Phenomenon and Passive vs. Active Components are provided by the Energy Efficiency equation (Mathematical Core of Sustainable Network Development). The script models PRIS (the power of one RIS element) as only 0.005 Watts, according to Passive vs. Active Components. This demonstrates that, in contrast to using an active relay, adding 64 RIS elements hardly raises the hardware power load of the base station. But according to the green curve phenomena, energy efficiency gradually decreases as more transmitted power (Pt) is added to the system. This is because data rate (R) only increases logarithmically while power consumption increases linearly.

The energy efficiency is calculated as

$$\eta_{EE}=\frac{R}{P_{total}}$$

where $\eta_{EE}$ is measured in bits/Joule, R is the achievable data rate in bits/s, and $P_{total}$ is the total consumed power in watts. The total power is expressed as

$$P_{total}=P_{tx}+P_{BS,c}+NP_{RIS}+P_{UE,c},$$

where $P_{tx}$ is the transmit power, $P_{BS,c}$ is the circuit power of the base station, $P_{RIS}$ is the average power consumption per RIS element, N is the number of RIS elements, and $P_{UE,c}$ is the user-equipment circuit power.

The RIS Advantage: The RIS curve will peak far higher than the non-RIS curve, demonstrating that modifying the propagation environment (RIS) is a better long-term approach to boosting network capacity than merely raising transmit power. Table 1 provides particular numerical values for the variables to create graphs in order to develop a MATLAB program 2020a to simulate and assess the sustainability of the Reconfigurable Intelligent Surface (RIS) network system. The equations’ mathematical notation is displayed in the Simulation Parameters table. Here is a thorough description of its significance, structure, and direct translation into MATLAB code.

The estimated power dissipation of each RIS element $P_{RIS}=0.005$ W represents the low average drive and bias power dissipation associated with each passive reflective element. Since the RIS is modeled as a passive reflective surface, this value does not reflect the radiated transmit power. Instead, it considers the electronic power required for phase state setup, bias circuitry, and gate-level control. The exact value may vary depending on RIS implementation, phase resolution, switching technology and controller architecture. Therefore, this parameter is treated as an assumption for the actual simulation and is included in the interpretation of the sensitivity of the green operating point.

### 7.1. Sustainable RIS System Model

In contemporary wireless networks, we often come across situations where buildings, trees, or interior walls obstruct the direct signal from a cell tower to a user. The User Equipment (UE) has a single antenna, much like a standard smartphone receiving a signal, while the Base Station (BS) utilizes M antennas to focus its transmission (beamforming). On the other hand, the RIS is a flat surface with N passive reflecting pieces that is mounted to a wall or other structure. These components are often composed of inexpensive printed materials that have the ability to change the phase of radio waves that strike them. Sustainable development and Reconfigurable Intelligent Surface (RIS) systems are closely related. Conventional methods of increasing wireless coverage typically entail deploying dozens of additional, power-hungry active relay nodes or brute-forcing the issue by increasing the transmit power from the Base Station (BS). This paradigm is reversed by RIS, which is in perfect harmony with the objectives of global sustainability.

A BS with M antennas, a single-antenna user equipment (UE), and a RIS with N passive reflecting elements make up a downlink Multiple-Input Single-Output (MISO) communication system. Equation (1) [6] can be used to express the signal received at the UE.

(1) $$y=\left(h_{d}^{H}+h_{r}^{H}\Theta G\right)ws+n$$

where

$h_{d}^{H}\;\in \;{∁}^{1xM}$ is the direct channel for the base station (BS) to the user’s equipment (UE);

$G\;\in \;{∁}^{NxM}$ is the channel matrix from the base station (BS) to the RIS;

$h_{r}^{H}\;\in \;{∁}^{1xN}$ is the direct channel from RIS to the user equipment (UE);

$w\;\in \;{∁}^{Mx1}$ is the active beamforming vectors applied at the BS;

s is the transmitted data symbol with $E\left[{\left|s\right|}^{2}\right]=1$;

$n\;\sim \;CN(0,\sigma^{2})$ is the additive White Gaussian Noise (AWGN) at the user equipment.

Θ is the diagonal reflection coefficient matrix of the RIS, defined as

$$\Theta =diag(e^{j\theta 1},e^{j\theta 2},e^{j\theta 3},\;.\;.\;.,\;e^{j\theta x})$$

where $\theta_{n}$ [0, 2π) correspond to the phase shift concerned by the n-th RIS element. RIS raises the Signal-to-Noise Ratio (SNR). Equation (2) illustrates a concept known as constructive interference at the receiver.

A base station with Mantenas, a passive *RIS* with *N* reflecting components, and single-antenna user equipment make up the downlink system under consideration. The direct *BS-UE*, *RIS-UE*, and *BS-RIS* channels are shown by $G\in C^{N\times M}$, $h_{r}\in C^{N\times 1}$, and $h_{d}\in C^{M\times 1}$, respectively. The RIS phase-shift matrix is stated as $\Theta =diag(\beta_{1}e^{j\theta_{1}},\beta_{2}e^{j\theta_{2}},...,\beta_{N}e^{j\theta_{N}})$, where $\beta_{n}\in [0,1]$ is the reflection amplitude and $\theta_{n}\in [0,2\pi )$ is the n-th reflecting element’s modifiable phase shift.

A reconfigurable intelligent surface (RIS) increases the signal-to-noise ratio (SNR) by cleverly adjusting the phase of the incoming signals at each of its reflecting elements This creates constructive interference because the reflected signals reach the receiver exactly in phase with each other.

(2) $$SNR=\frac{p_{t}{\left|h_{d+}h_{r}^{H}\Theta h_{sr}\right|}^{2}}{\sigma^{2}}$$

where

*P_t_* is the transmit power;

$h_{d}$ is the coefficient of direct communication between the transmitter (Tx) and receiver (Rx);

$h_{sr}\;\in \;{∁}^{Nx1}$ is the channel vector between the RIS and the Tx;

$h_{rd}\;\in \;{∁}^{Nx1}$ is the channel vector that connects the RIS to the Rx.

The Direct Path: The signal goes directly from the base station to the user equipment. However, this approach is frequently weak in the actual world due to obstacles like walls, trees, and buildings. The BS also sends waves in the direction of the RIS via the Reflected Path, also known as the RIS Path. These waves are reflected toward the UE by the RIS, which functions as a smart mirror.

The complex summation becomes a simple sum of real amplitudes since the phases are now completely aligned. The signals now stack rather than cancel each other out. Equation (3) for optimal SNR becomes:

(3) $${SNR}_{MAX}=\;\frac{p_{t}{\left|h_{d}+\sum_{n=1}^{N}h_{rd},n*h_{sr,n}\right|}^{2}}{\sigma^{2}}$$

Under ideal coherent combining, the RIS operates in the far-field region with continuous phase control, all reflected paths are phase-aligned at the receiver, the reflecting elements experience comparable path gains, and mutual coupling is ignored. This results in the well-known N^2^ received power scaling. Phase quantization, amplitude loss, imprecise Channel State Information (CSI), element coupling, mobility-induced phase variation, and controller latency can all deteriorate this ideal scaling in real-world applications. Thus, rather than being an absolute practical guarantee, the $N^{2}$ trend is regarded here as an upper-bound behavior employed for sustainability-oriented performance evaluation.

The total is included within a squared term in the blocked-path equation. This indicates that the power of the received signal scales in proportion to N^2^, which is the square of the number of reflecting elements. Constructive interference offers a significant SNR improvement over conventional relays because of this N^2^ scaling, which is the distinguishing feature of RIS technology.

**Figure 4:**
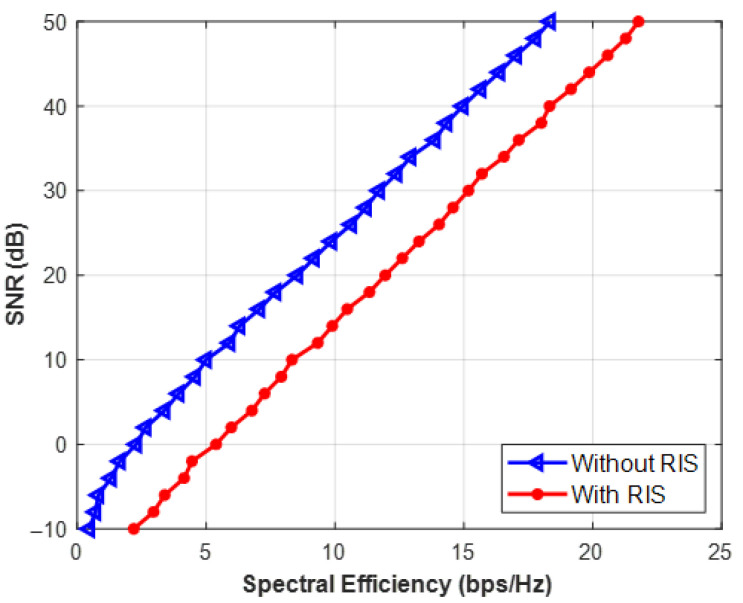
SNR vs. spectral efficiency for MISO System.

The data rate (Spectral Efficiency) of a regular network and one that uses a Reconfigurable Intelligent Surface (RIS) with eight reflecting elements is contrasted in Figure 4. There is a constant increase in the RIS curve. This demonstrates the fundamental “green” advantage of RIS. The network achieves a higher data rate without requiring the base station to emit additional raw radio frequency (RF) power into the environment by passively reflecting existing signals to get around obstructions.

The basic baseline of a Reconfigurable Intelligent Surface (RIS) is established in this illustration. It contrasts the data rate that a typical base station with four antennas can achieve with one that is assisted by a small RIS panel with eight reflecting elements. The network produces a highly concentrated beam of energy that is precisely aimed at the user by cleverly modifying the surroundings. This implies that in order to guarantee a solid connection, the Base Station does not need to squander electricity broadcasting signals in every direction. The current analysis assumes quasi-static channels under the Urban Micro (UMi) path-loss model for analytical tractability. This paper does not explicitly model handover jitter, mobility-induced Doppler shifts, or UAV/HAP trajectory dynamics.

**Figure 5:**
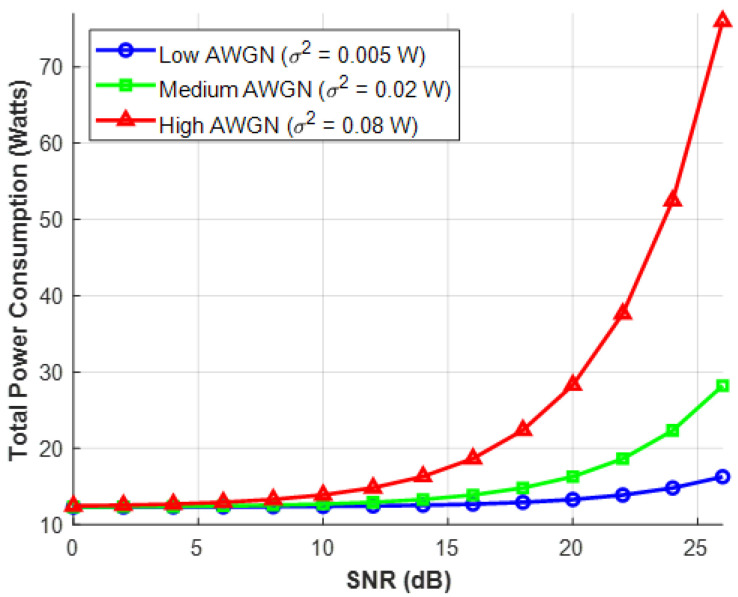
Total RIS System Power vs. SNR for Various AWGN Levels.

Additive White Gaussian Noise (AWGN) is used in Figure 5 to present environmental realism. It displays several power consumption curves that correspond to conditions with low, medium, and high noise levels. The Extensive Analysis: The ratio of signal to noise is known as SNR. The background noise (σ^2^) increases as a truck passes by or a microwave is turned on. The Base Station must dynamically multiply its broadcast power to overwhelm the new noise in order to retain the exact same SNR target. The angle of sustainability: This graph illustrates the environmental unsustainability of “brute-forcing” a signal. Adaptive modulation is implemented using this data in a green network technique. The network will tolerate a momentarily lower SNR, modestly lowering the user’s signal quality in order to save a significant amount of power, rather than using a lot of energy to combat a brief noise spike.

### 7.2. Sustainable Power Consumption Model

Equation (4) illustrates how the overall power consumption model must take into consideration both the transmitting power and the hardware dissipation to appropriately represent the network’s sustainability:

(4) $$P_{total}=\frac{1}{\mu }∥\overset{⃗}{w}{∥}^{2}+P_{BS}+P_{UE}+N\cdot P_{RIS}$$

where

$P_{total}$: The total power consumption of the entire communication system.

μ∈(0,1): BS’s power amplifier efficiency at the transmitter.

w: The active beamforming vector at the Base Station. The term $∥\overset{⃗}{w}{∥}^{2}$ (the squared norm of the vector) represents the actual RF (radio frequency) transmit power sent over the air.

$P_{BS}$: The power consumed by a base station (BS) just to operate its internal circuits, regardless of the transmit power.

$P_{UE}$: Static hardware power consumed by the user equipment (UE), which is the mobile device receiving the signal.

N: Total number of reflective elements on reconfigurable smart surface.

$P_{RIS}$: Hardware power consumed by the RIS reflector element. Multiply this by N to get the total power consumed by the entire RIS card.

The first EE goal was a fractional program that ratios the total power consumption to the achievable sum-rate. Adapted Equations (5)–(8), by the authors based on Dinkelbach’s fractional programming framework [6]. We use Dinkelbach’s transformation to separate the numerator and denominator in order to make this tractable. We convert the fractional objective into the parametric subtractive form given in Equation (5) by adding an auxiliary variable λ, which stands for optimal energy efficiency:

(5) $$F\left(w,\Theta ,\lambda \right)=B{log}_{2}\left(1+SNR\left(w,\Theta \right)\right)-\lambda P_{total}\left(w\right)\;$$

Accordingly, the optimizer iteratively looks for this function’s roots. Equation (6) formulates the new subtractive problem for a given iteration with a fixed λ as [6].

(6) $${Sub}_{p}\left({max}_{w,\Theta }\right)=B{log}_{2}\left(1+\frac{{\left|h_{d}^{H}+h_{r}^{H}\Theta G\right|}^{2}}{\sigma^{2}}\right)-\lambda \left(\frac{1}{\mu }{\left\|w\right\|}^{2}+P_{total}\right)$$

**Figure 6:**
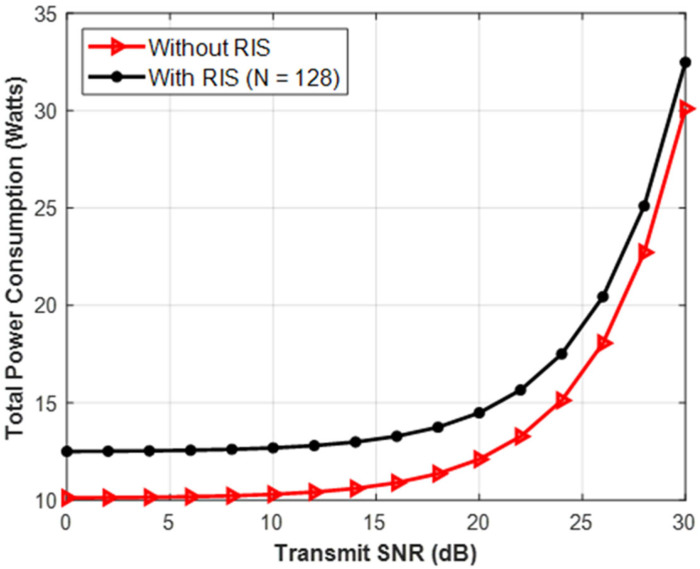
Total Power Consumption vs. Transmit SNR.

The denominator of the EE equation (P_total_) is isolated and plotted versus the SNR in Figure 6. It displays a curve with a relatively flat beginning and an exponential upward spike. The Extensive Analysis: The two components of total power are active transmission power (the RF energy blasted from the antennas) and static hardware power (the energy required simply to maintain the computer chips, sensors, and RIS controller turned on). The flat hardware power is dominant at low SNRs. However, every additional Watt of RF power required by a high SNR necessitates drawing two Watts from the electrical grid because telecom power amplifiers are usually only about 50% efficient. The carbon impact of “greedy” network designs is shown in the Sustainability Angle. Since the top 10% of SNR performance accounts for the majority of a cell tower’s power usage, it demonstrates that mobile carriers must cap their highest transmit power in order to meet net-zero emission requirements.

**Figure 7:**
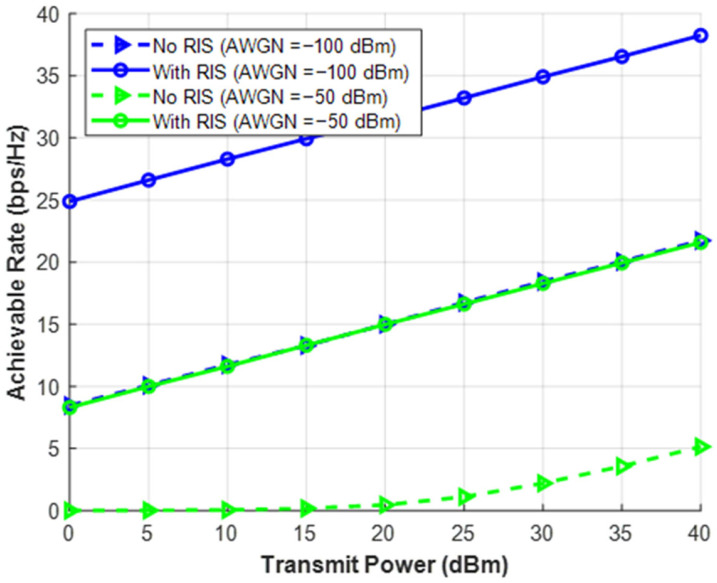
Total power consumption vs. transmits SNR.

All of this is brought together in Figure 7, which plots curve for various noise levels both with and without a RIS and displays the possible data rate against raw transmitting power. The Deep Dive: This storyline effectively demonstrates the “RIS Rescue Effect.” It demonstrates that 40 dBm of power may be needed to sustain a basic connection on an unassisted network buried in loud noise. That same noisy environment can accomplish high data speeds with a fraction of the electricity by implementing a RIS. The angle of sustainability: In the past, a telecom firm would construct a brand-new active cell tower or a powered relay station if an area had poor signal (high noise/blockage). These call for large electrical drawings, air conditioners, and substantial concrete infrastructure. This figure demonstrates how the coverage issue can be resolved with practically no additional footprint by adhering inexpensive, nearly zero-power, passive RIS panels to the fade of existing buildings.

### 7.3. Sustainable Energy Efficiency Maximization

The non-convex character of Energy Efficiency (EE) maximization is the main obstacle to developing Sustainable RIS Integration. The objective function combines the strict unit-modulus constraints of the passive elements with the active beamforming vector (w) at the Base Station (BS) and the passive phase-shift matrix (Θ) at the RIS. We use a two-tier mathematical framework that combines Alternating Optimization (AO) and Fractional Programming to methodically address issues. Alternating Optimization (AO) decomposes the non-convex joint optimization problem into two subproblems: optimizing the active beamforming vector while fixing RIS phase shifts and optimizing RIS phase shifts while fixing the beamforming vector. These steps are iteratively repeated until convergence. Equation (7) defines the maximum Energy Efficiency (ηEE) as the ratio of the total data rate to the total power consumed by the entire system:

(7) $${\eta EE}_{MAX}=\frac{B{log}_{2}(1+SNR)}{p_{total}}\;$$

Researchers must include the complete communication system, not just the RIS, while analyzing RIS for Energy Efficiency (EE), which is evaluated in bits/Joule. Equation (8) models a RIS-assisted system’s overall power consumption P_total_ as:

(8) $$P_{total}=\frac{p_{t}}{\mu }+P_{BS}+P_{UE}+P_{RIS\;\;}$$

where P_max_ is the highest transmit power permitted at the base station and B is the system bandwidth. Because the objective function and the unit-modulus constraints of the RIS elements are non-convex, addressing these calls for sophisticated methods like fractional programming and Alternating Optimization (AO). Through iterative convergence, AO delivers better energy efficiency than heuristic beamforming techniques, albeit at a larger computing complexity.

Alternating Optimization is used to address the non-convex energy-efficiency maximization issue. Every iteration optimizes the active transmit beamforming vector while maintaining a fixed RIS phase-shift matrix. Next, the beamforming vector is fixed while the RIS phase shifts are updated. Until the difference in energy efficiency between two successive iterations is less than a predetermined tolerance, these two processes are repeated. The energy-efficiency and Green Operating Point curves shown in the results section are then generated using the optimal beamforming and RIS phase configuration.

**Figure 8:**
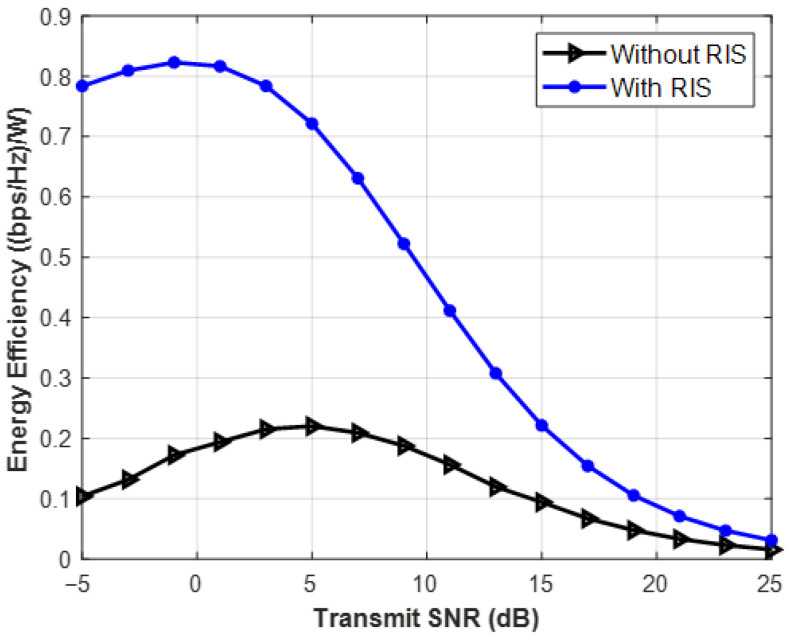
Energy Efficiency vs. Transmit SNR.

Energy Efficiency (measured in bits per Joule) is plotted against the desired Signal-to-Noise Ratio (SNR) in Figure 8. A clear bell curve with a recognizable apex is the resultant graph. The Extensive Analysis: The formula controls energy efficiency. This graph compares the SNR to the Energy Efficiency (bits transmitted per Joule of energy). It creates a bell-shaped curve with a clear peak. In terms of green communications, this is the goal. Here, the previous approach of “maximizing the SNR at all costs” ought to be abandoned. This curve’s peak is known as the “Green Operating Point.” This SNR threshold will never be exceeded by sustainable network protocols, guaranteeing the highest data yield per unit of electricity extracted from the power grid.

**Figure 9:**
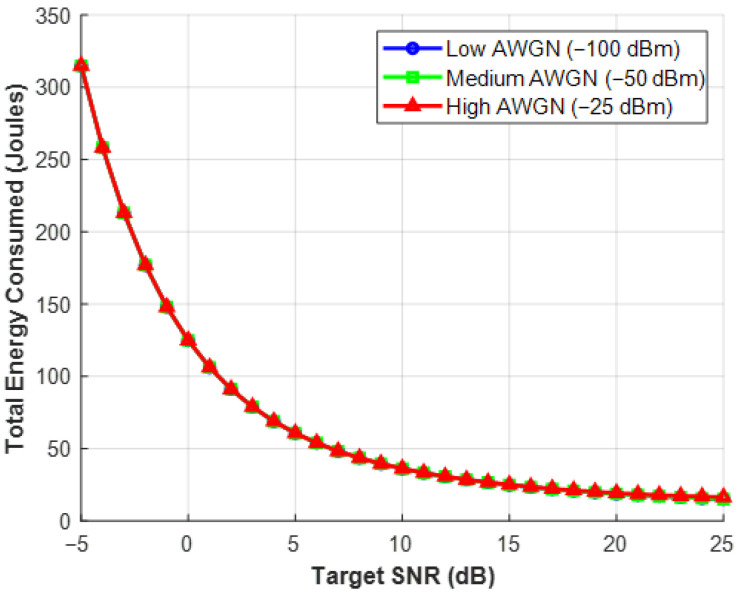
Total Energy to Transmit 10 Mbits vs. SNR (RIS System).

This picture displays energy (the total volume of battery consumed to accomplish a given job, such delivering a 10 Mbit file), whereas Figure 9 displays power (the pace of battery drain). It creates a striking bowl curve in the shape of a U. The Extensive Analysis: A U-shaped or bowl-shaped curve is created when total energy is calculated by multiplying power by time. Transmitting too quickly causes the power amplifier to run out of battery, while transmitting too slowly causes hardware to remain powered on for an extended period of time and waste energy. In order to maximize battery life and reduce charging cycles, sustainable IoT and 6G devices will be configured to always function at the precise bottom of this “U”.

Due to static hardware drain, the Base Station and the user’s phone typically need to stay turned on.

## 8. Discussion

In conventional wireless networks, increasing the transmitted power in the system is typically necessary to achieve a high Signal-to-Noise Ratio (SNR). The objective of sustainable wireless communication, on the other hand, is completely different: networks must provide dependable SNR and high data rates while drastically reducing energy consumption. Researchers are moving away from just optimizing SNR because base stations use the majority of the power in a cellular network. Rather, they optimize for Energy Efficiency (EE), which is the ratio of the data rate to the total power consumption, and treat SNR as a baseline requirement.

### 8.1. The Green Operating Point and the Bell Curve Phenomenon

The simulation findings clearly show that optimizing the Signal-to-Noise Ratio (SNR) is no longer a practical approach for long-term 6G networks. The network’s EE creates a clear bell curve, as seen in the Energy Efficiency against Transmit SNR charts. The static hardware power (P_BS_ + P_UE_) predominates at low SNRs, leading to subpar bits-per-Joule performance. Data rates growth logarithmically as transmitting power rises, yet the amount of RF power needed from the grid increases linearly (made worse by conventional power amplifiers’ 50% inefficiency).

At the top of the curve, this discrepancy establishes a “Green Operating Point” that can be verified numerically. A catastrophic decline in energy efficiency occurs when the network’s transmit power is pushed over this peak to achieve a slightly higher SNR. Future sustainable protocols must be dynamically bound to never surpass this peak, exchanging substantial grid power savings for needless excessive data rates.

### 8.2. The N^2^ Power Scaling Advantage of RIS

The N^2^ power scaling law is the key observation confirming RIS’s durability. The RIS phase changes are synchronized to create perfect constructive interference at the receiver using the Alternating Optimization technique. As a result, the power consumption of the RIS array only scales linearly (N × 0.005 W), although the received signal power scales according to the square of the number of reflecting elements (N^2^).

This demonstrates that increasing the size of a passive RIS array from 8 to 64 elements results in a significant quadratic improvement in the quality of the received signal while only increasing the total hardware power burden by 0.28 Watts. On the other hand, boosting the Base Station transmit power or using active relays to achieve the same SNR gain would necessitate multi-watt increases, requiring a substantial increase in raw energy from the electrical grid.

### 8.3. Energy Volume Trade-Offs for Sustainable IoT

Lastly, the system model shows a severe U-shaped consumption curve when examining the entire amount of battery energy used to send a fixed file size (such as a 10 Mbit payload). The baseline hardware must stay switched on for a long time when transmitting too slowly (low power), which wastes energy due to static leakage. Transmitting too fast (high power) causes the power amplifier to waste energy. The bottom of this U-curve becomes deeper and wider when a RIS is integrated. The RIS ensures a vital foundation for the sustainable lifespan of billions of future IoT devices by passively increasing the channel quality, allowing the user equipment to transmit the 10 Mbit file at an appropriate pace that reduces both active transmissions drain and static “awake time” drain.

### 8.4. RIS System Analysis in Wireless Communication

RIS changes the physical wireless environment from a passive barrier to an active, programmable network element by dynamically changing the phase and amplitude of electromagnetic waves. This enhances wireless communication.

#### 8.4.1. Strengths (Internal Advantages)

Since the majority of conventional RIS components are either completely passive or semi-passive, RIS provided Highly Energy-Efficient (Green Communications). They significantly lower the carbon footprint of network densification by reflecting and steering current signals without the need for power-hungry radio frequency (RF) links or power amplifiers.

Installing a passive RIS panel on an inside wall or building facade is far less expensive than installing a conventional active base station or small cell because of the Cost-Effective Coverage Extension. Additionally, Signal Enhancement in Harsh Bands offers a highly effective way to overcome the significant propagation losses associated with high-frequency bands like sub-Terahertz (sub-THz) and millimeter-wave (mmWave) and improve the link budget. For the system flexibility and form factor, the Meta surfaces can be made to be incredibly thin, conformal, and even optically clear (transparent glass RIS for windows, for example), which enables them to mix in perfectly with urban design.

#### 8.4.2. Comparison with the Existing Work

Strong signal strength is only one aspect of sustainability in telecommunications; other requirements include minimizing hardware manufacturing footprints, lowering ongoing grid power use, and maintaining low computing needs. Table 2 indicates a comparative Analysis of RIS with Existing Work”. It evaluates three different approaches to Reconfigurable Intelligent Surface (RIS) communication systems based on their underlying methods, Energy Efficiency (EE) gains, and overall system complexity.

This table shows that although an Active RIS has the highest absolute energy efficiency, it requires a high level of computational and hardware complexity. By employing algorithmic techniques (AO and GOP), the suggested approach achieves high, sustainable energy efficiency while maintaining a reasonable medium level of system complexity.

#### 8.4.3. Opportunities (External Market Potential)

The 6G Imperative: Prominent industry analysts concur that signal attenuation will make the deployment of 6G, which aims for 100 Gbps^−1^ Tbps at sub-THz frequencies, economically unfeasible in the absence of RIS.

Advanced RIS Architectures: Massive new capabilities are made possible by the switch from basic reflective panels to advanced architectures like STAR-RIS (Simultaneous Transmitting and Reflecting), Active RIS (which includes low-power amplifiers to overwhelm the double-path loss), and Elsewhere Diagonal RIS (BD-RIS). For Zero-Energy Devices (ZED) & SWIPT, the RIS can serve as an energy hub by remotely charging battery-free IoT devices and sensors using Simultaneous Wireless Information and Power Transfer (SWIPT). However, the integration with Non-Terrestrial Networks (NTN) shows that the RIS can be mounted on High-Altitude Platform Stations (HAPS) or Unmanned Aerial Vehicles (UAVs) to provide dynamic, three-dimensional network coverage for rural or disaster-affected areas.

### 8.5. Practical Limitations and Deployment Conditions

Imperfect CSI can change the Green Operating Point and decrease phase-alignment accuracy in real-world RIS systems. Constructive interference stability may be weakened by the mobility of UAV-mounted RIS and handover-induced phase jitter. Additionally, quantifiable phase shifts, controller latency, and amplitude-dependent reflection losses could be problems for practical RIS hardware.

Under ideal propagation conditions, RIS-assisted transmission can increase coverage and energy efficiency, although these benefits are not always realized. When the direct BS–UE channel is blocked or significantly attenuated, and when the RIS is placed in a geometrically advantageous location that offers strong BS–RIS and RIS–UE links, the benefits are anticipated to be substantial. On the other hand, if the direct link is already strong, if the RIS is not positioned correctly, if phase alignment is off, or if user mobility results in rapid channel variation, the advantages might be restricted. Finite phase resolution, phase-tuning errors, controller delay, CSI acquisition overhead, element coupling, and reflection-amplitude losses all have an impact on practical implementation. Therefore, rather than being universal guarantees for all network deployments, the results should be considered as sustainability-oriented performance predictions under restricted modeling assumptions.

The current study contains several shortcomings. First, the channel model does not specifically account for high-mobility users, Doppler effects, or phase instability caused by handover because it assumes quasi-static propagation. Second, idealistic formulation is used to model RIS phase control, while hardware nonlinearity, controller delay, reflection loss, and finite phase resolution all have an impact on real-world implementations. Third, the chosen RIS power-consumption model may differ depending on the hardware platform and is simplified. Fourth, a single-user baseline is the focus of existing architecture.

### 8.6. Considerations for Future Research

Therefore, robust Green Operating Point optimization under faulty CSI, mobility-aware RIS phase tracking, hardware-in-the-loop validation, finite-resolution phase control, and multi-user/multi-RIS deployment situations should be the focus of future research. The essay identifies several essential avenues for further research in order to overcome these limitations:

- Strong Green Operating Point optimization techniques that can work well in the face of faulty CSI need to be researched.
- Mobility-aware RIS phase tracking and finite-resolution phase control must be considered in future research.
- Hardware-in-the-loop validation and the investigation of increasingly intricate multi-user and multi-RIS deployment situations are needed.

## 9. Contributions

The development of a Green Operating Point methodology for RIS-assisted mobile networks is this work’s primary contribution. The suggested methodology determines the most energy-efficient operating region by connecting attainable rate, transmit power, RIS hardware power, and fixed-payload energy consumption. This is different from traditional research, which focuses on maximizing received signal strength or spectral efficiency without clearly defining a feasible sustainability threshold. The Key Contributions stated in Consecutive points:

- The Green Operating Point is a verifiable threshold that restricts base stations transmitting power. Since grid power consumption scales linearly and data speeds only scale logarithmically, limiting electricity at this precise peak eliminates significant energy waste while maintaining the necessary Quality of Service.
- **IoT Volumetric Energy Trade-off:** The study determines the precise bottom of a rigid, U-shaped energy consumption curve by calculating the total energy (in Joules) needed to transmit a fixed data payload (e.g., 10 Mbits). This offers a guide for optimizing the battery life of billions of IoT devices in the future.
- **Quantification of the N^2^ Power Scaling Law:** The study demonstrates that received signal power scales proportionately to the square of the reflecting elements N^2^ using an Alternating Optimization framework. It shows that in extremely obstructed and loud situations, 40 dBm of raw active transmit power can be successfully replaced by adding 64 RIS pieces, which demand a mere 0.32 Watts of hardware power.
- **Two-Level Mathematical Structure for 6G Sustainability:** Using Dinkelbach’s transformation and Alternating Optimization, the authors developed a cooperative resource allocation model. Net-zero network deployments are made possible by the framework’s effective solution of the extremely non-convex EE maximization issue, which separates active base station beamforming from the severe unit-modulus limitations of passive RIS phase shifts.

## 10. Conclusions

In conclusion, significant change from merely improving the Signal-to-Noise Ratio (SNR) to optimizing total Energy Efficiency (EE) is necessary to achieve sustainable development in networks beyond 5G and 6G. This study unequivocally shows that Reconfigurable Intelligent Surfaces (RIS) provide a very energy-efficient way to get around indoor and urban blocks without the harsh environmental consequences of conventional active relays. According to system evaluations, most of a cell tower’s power consumption is attributed to the top 10% of SNR performance. Every additional Watt of RF power required by a high SNR necessitates drawing two Watts from the electrical grid due to the roughly 50% inefficiency of conventional telecom power amplifiers. Brute-forcing signal strength to support future 6G target speeds of 100 Gbps to 1 Tbps at sub-THz frequencies is not viable from an economic and environmental standpoint. Through N2 power scaling, where individual metamaterial pieces need only 0.005 Watts, networks can achieve enormous capacity improvements by utilizing RIS. RIS allows network devices to function precisely at the lowest point of the energy consumption curve, whether it is analyzing the total volume of battery energy drained to transmit a 10 Mbit file or maintaining robust coverage in high-noise environments that would otherwise require 40 dBm of transmit power. In the end, adding passive, low-power panels with 8-to-64 reflecting components to power-hungry antenna arrays turns out to be a crucial, economical method for enabling next-generation deployments while achieving global net-zero emissions. In addition to experimental validation using real-world RIS hardware, future work will expand the suggested framework to examine robust, network-level optimization and dynamic mobility-aware RIS phase adaptation under faulty CSI and multi-user interference.

## Figures and Tables

**Table 1:** Simulation Parameters.

Parameter	Symbol	Value	Unit	Justification
Number of BS antennas	M	4 to 16	—	Simulation hypothesis
Number of RIS elements	N	8 to 256	—	RIS-size sensitivity study
RIS element power	P_RIS_	0.006	W	Average control
BS power	P_BS_	10	W	Base station power
UE power	P_UE_	10	mW	User equipment power model
Carrier frequency	*f_c_*	30–60	GHz	6G/UMi simulation setup
Bandwidth	(B)	10	MHz	Rate calculation
Path-loss model	P_Ld_	3	GPP UMi	Urban microcell scenario
Channel condition	—	Quasi-static	—	Baseline analytical tractability
Noise Power Spectral Density	No	−175	dBm/Hz	Thermal-noise model
Algorithm Convergence Threshold	ϵ	10^−4^	-	Small positive scalar
Power Amplifier Efficiency	μ	50	%	Optimal value

**Table 2:** Comparative Analysis of RIS with Existing Work.

Work	Method	EE Gain	Complexity
Huang et al. [13]	Joint Beamforming	High	High
Active RIS	Amplified Reflection	Very High	Very High
Proposed	Passive RIS + AO + GOP	High + Sustainable	Medium

## Data Availability

All data supporting the findings of this study are already included within the article.
